# Dispatch from the field: ecology of ground-web-building spiders with description of a new species (Araneae, Symphytognathidae)

**DOI:** 10.3897/BDJ.2.e1076

**Published:** 2014-03-26

**Authors:** Jeremy A. Miller, Menno Schilthuizen, Jennie Lilliendahl Burmester, Lot van der Graaf, Vincent Merckx, Merlijn Jocqué, Paul Joseph Antonius Kessler, Tom Maurice Fayle, Thijmen Breeschoten, Regi Broeren, Roderick Bouman, Wan-Ji Chua, Frida Feijen, Tanita Fermont, Kevin Groen, Marvin Groen, Nicolaas Johannes Cornelis Kil, Henrica Allegonda de Laat, Michelangelo Sergio Moerland, Carole Moncoquet, Elisa Panjang, Amelia Joyce Philip, Rebecca Roca-Eriksen, Bastiaan Rooduijn, Marit van Santen, Violet Swakman, Meaghan N. Evans, Luke J. Evans, Kieran Love, Sarah H Joscelyne, Anya Victoria Tober, Hannah F. Wilson, Laurentius N. Ambu, Benoit Goossens

**Affiliations:** † Naturalis Biodiversity Center, Leiden, Netherlands; ‡ Plazi, Bern, Switzerland; § Universiti Malaysia Sabah, Kota Kinabalu, Malaysia; | University of Copenhagen, Copenhagen, Denmark; ¶ Delft University, Delft, Netherlands; # Rutgers, the State University of New Jersey, Newark, United States of America; †† Hortus Botanicus, Leiden University, Leiden, Netherlands; ‡‡ Faculty of Science, University of South Bohemia and Institute of Entomology, Biology Centre of Czech Academy of Sciences, České Budějovice, Czech Republic; §§ Forest Ecology and Conservation Group, Imperial College London, London, United Kingdom; || Leiden University, Leiden, Netherlands; ¶¶ Antwerp University, Antwerp, Belgium; ## Utrecht University, Utrecht, Netherlands; ††† Wageningen University, Wageningen, Netherlands; ‡‡‡ University of Amsterdam, Amsterdam, Netherlands; §§§ Danau Girang Field Centre, c/o Sabah Wildlife Department, Kota Kinabalu, Malaysia; ||| Cardiff University, Cardiff, United Kingdom; ¶¶¶ Sabah Wildlife Department, Kota Kinabalu, Malaysia

**Keywords:** Borneo, *
Crassignatha
*, disturbance, inundation, oil palm plantation, riparian forest, riverine forest, tropical field course

## Abstract

*Crassignatha danaugirangensis* sp. n. (Araneae: Symphytognathidae) was discovered during a tropical ecology field course held at the Danau Girang Field Centre in Sabah, Malaysia. A taxonomic description and accompanying ecological study were completed as course activities. To assess the ecology of this species, which belongs to the ground-web-building spider community, three habitat types were surveyed: riparian forest, recently inundated riverine forest, and oil palm plantation. *Crassignatha danaugirangensis* sp. n. is the most abundant ground-web-building spider species in riparian forest; it is rare or absent from the recently inundated forest and was not found in a nearby oil palm plantation. The availability of this taxonomic description may help facilitate the accumulation of data about this species and the role of inundated riverine forest in shaping invertebrate communities.

## Introduction

*Crassignatha* Wunderlich is a genus of six-eyed micro-orbweaving spiders on the order of 1 mm in total body length. The genus was established to accommodate a single male specimen collected at Fraser's Hill (Bukit Fraser in Malay), a forest reserve in Pahang, Malaysia (Wunderlich 1995). Miller et al. (2009) described eight additional species of *Crassignatha* collected in the course of a survey of the Gaoligongshan in western Yunnan, China. Only one of these species was a singleton and most species were known from 7–20 adult specimens, suggesting that they can be abundant. But few species were collected at more than one locality and the rate of species turnover in the Gaoligongshan appeared to be on the order of 50–100 km.

Students participating in a two-week tropical ecology field course offered by the Naturalis Biodiversity Center and hosted by the Danau Girang Field Centre (DGFC) in Sabah, Malaysia, encountered a species of *Crassignatha* in the course of their studies. Without doubt, undescribed arthropod species abound in the vicinity of any tropical field station, but being able to recognize which species are new requires expertise and access to literature and reference collections not often available in such remote places. In this case, the corresponding author was able to determine that the Danau Girang *Crassignatha* was new, and since only two taxonomic papers have treated members of this genus, the collection of reference literature was soon complete. Students and lecturers participating in the course, along with members of the field station scientific staff, resolved to describe this species and investigate its ecology using the resources available at the field station and submit their results in the form of a manuscript before the end of the course.

DGFC is a research station on the Kinabatangan River in Sabah, Malaysia. The Kinabatangan River floods periodically, inundating the low-lying parts of the forest. Beyond the protected forest areas, the dominant land use is oil palm plantation. Within Danau Girang, four quarter hectare plots have been established as permanent botanical plots. Within these plots, all trees have been taxonomically identified (as far as possible) and labeled with a unique number. As the course arrived at DGFC, the water in the inundated forest was just in the process of receding.

## Materials and methods

Spider samples were taken from DGFC's four permanent botanical plots and from the nearby Hillco Estate oil palm plantation (5.415°N 118.016°E) just across the Kinabatangan River. Two of the botanical plots (2 and 3) are subject to regular inundation (1–3 times per year) while the two remaining plots (1 and 4) are flooded only during extraordinary weather events (approximately once in 6–7 years). Despite inquiries, we were unable to determine details of the oil palm plantation history and management. Trees ranged from 2–3 m in height and we witnessed workers applying an unknown chemical.

Within the DGFC permanent botanical plots, 4–6 points were randomly selected; eight points from a block of oil palm plantation also were selected randomly. At each point, 1 m^2^ was dusted for spider webs 0–10 cm above the ground using a corn starch puffer (Carico 1977) and as many spiders as possible were collected using a pooter. In the oil palm plantation, additional plotless sampling was conducted. All adult spiders from the plot sampling were counted and sorted; the plotless sample was checked for the presence of *Crassignatha*.

### Laboratory methods

The taxonomic description was completed at the laboratory facilities of the Danau Girang Field Centre. Photographs were taken using an iPhone 4 through the ocular lens of a Leica Zoom 2000 stereomicroscope and an Omax compound microscope. Specimens were positioned for photography under the stereomicroscope using cotton wool. The vulva and male leg II were slide mounted and cleared for examination in palm oil.

All *Crassignatha* specimens have been deposited at the Universiti Malaysia Sabah's Institute for Tropical Biology and Conservation, Borneensis.

## Taxon treatments

### Crassignatha

Wunderlich, 1995

Crassignatha Wunderlich, 1995 – Wunderlich 1995: 546; Miller et al. 2009: 68.
Crassignatha Crassignatha haeneli Wunderlich, 1995

### Crassignatha danaugirangensis sp. n.

urn:lsid:zoobank.org:act:EDB4926E-0CBB-448F-B657-10373F6FD69F

#### Materials

- **Type status:** Holotype. **Occurrence:** catalogNumber: 20140304.1:57H; recordedBy: J. Miller, C.M. van der Graaf, C. Burmester; individualCount: 1; sex: 1 male; **Taxon:** scientificName: Crassignatha danaugirangensis; order: Araneae; family: Symphytognathidae; genus: Crassignatha; specificEpithet: danaugirangensis; taxonRank: species; scientificNameAuthorship: Miller et al. 2014; **Location:** island: Borneo; country: Malaysia; stateProvince: Sabah; locality: Danau Girang Field Centre, plot 1; verbatimCoordinates: 5°24.75'N 118°2.35'E; decimalLatitude: 5.4125; decimalLongitude: 118.0392; **Event:** samplingProtocol: dusting for webs; eventDate: 2014-03-04; **Record Level:** institutionID: Universiti Malaysia Sabah; collectionID: Institute for Tropical Biology and Conservation, Borneensis; institutionCode: UMS; collectionCode: BORN; basisOfRecord: PreservedSpecimen
- **Type status:** Paratype. **Occurrence:** catalogNumber: 20140304.1:57; recordedBy: J. Miller, C.M. van der Graaf, C. Burmester; individualCount: 4; sex: 4 females; **Taxon:** scientificName: Crassignatha danaugirangensis; order: Araneae; family: Symphytognathidae; genus: Crassignatha; specificEpithet: danaugirangensis; taxonRank: species; scientificNameAuthorship: Miller et al. 2014; **Location:** island: Borneo; country: Malaysia; stateProvince: Sabah; locality: Danau Girang Field Centre, plot 1; verbatimCoordinates: 5°24.75'N 118°2.35'E; decimalLatitude: 5.4125; decimalLongitude: 118.0392; **Event:** samplingProtocol: dusting for webs; eventDate: 2014-03-04; **Record Level:** institutionID: Universiti Malaysia Sabah; collectionID: Institute for Tropical Biology and Conservation, Borneensis; institutionCode: UMS; collectionCode: BORN; basisOfRecord: PreservedSpecimen
- **Type status:** Paratype. **Occurrence:** catalogNumber: 20140301.1:59; recordedBy: J. Miller, C.M. van der Graaf, C. Burmester; individualCount: 8; sex: 2 males, 3 females, 3 juveniles; **Taxon:** scientificName: Crassignatha danaugirangensis; order: Araneae; family: Symphytognathidae; genus: Crassignatha; specificEpithet: danaugirangensis; taxonRank: species; scientificNameAuthorship: Miller et al. 2014; **Location:** island: Borneo; country: Malaysia; stateProvince: Sabah; locality: Danau Girang Field Centre, plot 1; verbatimCoordinates: 5°24.75'N 118°2.35'E; decimalLatitude: 5.4125; decimalLongitude: 118.0392; **Event:** samplingProtocol: dusting for webs; eventDate: 2014-03-01; **Record Level:** institutionID: Universiti Malaysia Sabah; collectionID: Institute for Tropical Biology and Conservation, Borneensis; institutionCode: UMS; collectionCode: BORN; basisOfRecord: PreservedSpecimen
- **Type status:** Paratype. **Occurrence:** catalogNumber: 20140301.1:113; recordedBy: J. Miller, C.M. van der Graaf, C. Burmester; individualCount: 12; sex: 5 males, 3 females, 4 juveniles; **Taxon:** scientificName: Crassignatha danaugirangensis; order: Araneae; family: Symphytognathidae; genus: Crassignatha; specificEpithet: danaugirangensis; taxonRank: species; scientificNameAuthorship: Miller et al. 2014; **Location:** island: Borneo; country: Malaysia; stateProvince: Sabah; locality: Danau Girang Field Centre, plot 1; verbatimCoordinates: 5°24.75'N 118°2.35'E; decimalLatitude: 5.4125; decimalLongitude: 118.0392; **Event:** samplingProtocol: dusting for webs; eventDate: 2014-03-01; **Record Level:** institutionID: Universiti Malaysia Sabah; collectionID: Institute for Tropical Biology and Conservation, Borneensis; institutionCode: UMS; collectionCode: BORN; basisOfRecord: PreservedSpecimen
- **Type status:** Paratype. **Occurrence:** catalogNumber: 20140301.1:121; recordedBy: J. Miller, C.M. van der Graaf, C. Burmester; individualCount: 2; sex: 2 females; **Taxon:** scientificName: Crassignatha danaugirangensis; order: Araneae; family: Symphytognathidae; genus: Crassignatha; specificEpithet: danaugirangensis; taxonRank: species; scientificNameAuthorship: Miller et al. 2014; **Location:** island: Borneo; country: Malaysia; stateProvince: Sabah; locality: Danau Girang Field Centre, plot 1; verbatimCoordinates: 5°24.75'N 118°2.35'E; decimalLatitude: 5.4125; decimalLongitude: 118.0392; **Event:** samplingProtocol: dusting for webs; eventDate: 2014-03-01; **Record Level:** institutionID: Universiti Malaysia Sabah; collectionID: Institute for Tropical Biology and Conservation, Borneensis; institutionCode: UMS; collectionCode: BORN; basisOfRecord: PreservedSpecimen
- **Type status:** Paratype. **Occurrence:** catalogNumber: 20140301.1:60; recordedBy: J. Miller, C.M. van der Graaf, C. Burmester; individualCount: 2; sex: 2 females; **Taxon:** scientificName: Crassignatha danaugirangensis; order: Araneae; family: Symphytognathidae; genus: Crassignatha; specificEpithet: danaugirangensis; taxonRank: species; scientificNameAuthorship: Miller et al. 2014; **Location:** island: Borneo; country: Malaysia; stateProvince: Sabah; locality: Danau Girang Field Centre, plot 1; verbatimCoordinates: 5°24.75'N 118°2.35'E; decimalLatitude: 5.4125; decimalLongitude: 118.0392; **Event:** samplingProtocol: dusting for webs; eventDate: 2014-03-01; **Record Level:** institutionID: Universiti Malaysia Sabah; collectionID: Institute for Tropical Biology and Conservation, Borneensis; institutionCode: UMS; collectionCode: BORN; basisOfRecord: PreservedSpecimen
- **Type status:** Paratype. **Occurrence:** catalogNumber: 20140301.1:100; recordedBy: J. Miller, C.M. van der Graaf, C. Burmester; individualCount: 7; sex: 2 males, 5 females; **Taxon:** scientificName: Crassignatha danaugirangensis; order: Araneae; family: Symphytognathidae; genus: Crassignatha; specificEpithet: danaugirangensis; taxonRank: species; scientificNameAuthorship: Miller et al. 2014; **Location:** island: Borneo; country: Malaysia; stateProvince: Sabah; locality: Danau Girang Field Centre, plot 1; verbatimCoordinates: 5°24.75'N 118°2.35'E; decimalLatitude: 5.4125; decimalLongitude: 118.0392; **Event:** samplingProtocol: dusting for webs; eventDate: 2014-03-01; **Record Level:** institutionID: Universiti Malaysia Sabah; collectionID: Institute for Tropical Biology and Conservation, Borneensis; institutionCode: UMS; collectionCode: BORN; basisOfRecord: PreservedSpecimen
- **Type status:** Paratype. **Occurrence:** catalogNumber: 20140301.1:104; recordedBy: J. Miller, C.M. van der Graaf, C. Burmester; individualCount: 7; sex: 1 male, 6 females; **Taxon:** scientificName: Crassignatha danaugirangensis; order: Araneae; family: Symphytognathidae; genus: Crassignatha; specificEpithet: danaugirangensis; taxonRank: species; scientificNameAuthorship: Miller et al. 2014; **Location:** island: Borneo; country: Malaysia; stateProvince: Sabah; locality: Danau Girang Field Centre, plot 1; verbatimCoordinates: 5°24.75'N 118°2.35'E; decimalLatitude: 5.4125; decimalLongitude: 118.0392; **Event:** samplingProtocol: dusting for webs; eventDate: 2014-03-01; **Record Level:** institutionID: Universiti Malaysia Sabah; collectionID: Institute for Tropical Biology and Conservation, Borneensis; institutionCode: UMS; collectionCode: BORN; basisOfRecord: PreservedSpecimen
- **Type status:** Paratype. **Occurrence:** catalogNumber: 20140225F2; recordedBy: J. Miller, C.M. van der Graaf, C. Burmester; individualCount: 2; sex: 2 females; **Taxon:** scientificName: Crassignatha danaugirangensis; order: Araneae; family: Symphytognathidae; genus: Crassignatha; specificEpithet: danaugirangensis; taxonRank: species; scientificNameAuthorship: Miller et al. 2014; **Location:** island: Borneo; country: Malaysia; stateProvince: Sabah; locality: Danau Girang Field Centre, Mallotus trail; verbatimCoordinates: 5°25'N 118°2.08'E; decimalLatitude: 5.4666; decimalLongitude: 118.0392; **Event:** samplingProtocol: dusting for webs; eventDate: 2014-02-25; **Record Level:** institutionID: Universiti Malaysia Sabah; collectionID: Institute for Tropical Biology and Conservation, Borneensis; institutionCode: UMS; collectionCode: BORN; basisOfRecord: PreservedSpecimen
- **Type status:** Paratype. **Occurrence:** catalogNumber: 20140225M1; recordedBy: J. Miller, C.M. van der Graaf, C. Burmester; individualCount: 1; sex: 1 male; **Taxon:** scientificName: Crassignatha danaugirangensis; order: Araneae; family: Symphytognathidae; genus: Crassignatha; specificEpithet: danaugirangensis; taxonRank: species; scientificNameAuthorship: Miller et al. 2014; **Location:** island: Borneo; country: Malaysia; stateProvince: Sabah; locality: Danau Girang Field Centre, Mallotus trail; verbatimCoordinates: 5°25'N 118°2.08'E; decimalLatitude: 5.4666; decimalLongitude: 118.0347; **Event:** samplingProtocol: dusting for webs; eventDate: 2014-02-25; **Record Level:** institutionID: Universiti Malaysia Sabah; collectionID: Institute for Tropical Biology and Conservation, Borneensis; institutionCode: UMS; collectionCode: BORN; basisOfRecord: PreservedSpecimen
- **Type status:** Paratype. **Occurrence:** catalogNumber: 20140304.1:104; recordedBy: J. Miller, C.M. van der Graaf, C. Burmester; individualCount: 16; sex: 4 males, 10 females, 2 juveniles; **Taxon:** scientificName: Crassignatha danaugirangensis; order: Araneae; family: Symphytognathidae; genus: Crassignatha; specificEpithet: danaugirangensis; taxonRank: species; scientificNameAuthorship: Miller et al. 2014; **Location:** island: Borneo; country: Malaysia; stateProvince: Sabah; locality: Danau Girang Field Centre, plot 1; verbatimCoordinates: 5°24.75'N 118°2.35'E; decimalLatitude: 5.4125; decimalLongitude: 118.0392; **Event:** samplingProtocol: dusting for webs; eventDate: 2014-03-01; **Record Level:** institutionID: Universiti Malaysia Sabah; collectionID: Institute for Tropical Biology and Conservation, Borneensis; institutionCode: UMS; collectionCode: BORN; basisOfRecord: PreservedSpecimen
- **Type status:** Paratype. **Occurrence:** catalogNumber: 20140302.4:50; recordedBy: J. Miller, C.M. van der Graaf, C. Burmester; individualCount: 7; sex: 2 males, 5 females; **Taxon:** scientificName: Crassignatha danaugirangensis; order: Araneae; family: Symphytognathidae; genus: Crassignatha; specificEpithet: danaugirangensis; taxonRank: species; scientificNameAuthorship: Miller et al. 2014; **Location:** island: Borneo; country: Malaysia; stateProvince: Sabah; locality: Danau Girang Field Centre, plot 4; verbatimCoordinates: 5°24.5'N 118°2.4'E; decimalLatitude: 5.4083; decimalLongitude: 118.04; **Event:** samplingProtocol: dusting for webs; eventDate: 2014-03-02; **Record Level:** institutionID: Universiti Malaysia Sabah; collectionID: Institute for Tropical Biology and Conservation, Borneensis; institutionCode: UMS; collectionCode: BORN; basisOfRecord: PreservedSpecimen

#### Description

Coloration and gross somatic morphology as in Fig. 1a, b, c, d, e. Six eyes in three diads. Carapace dark brown, rough texture, raised in male (Fig. 1d). Sternum brown. Legs orange, femora I and II slightly swollen basally in female. Patellae each with a dorsal macroseta. Dorsal tibial macrosetae 2-2-1-1, tibia I with prolateral macroseta. Male tibia II clasping spur a single ventral macroseta, male metatarsus II shape unmodified (Fig. 1f, contrast with Miller et al. 2009, fig. 80E). Abdomen ovoid in dorsal view, subtriangular in lateral view with spinnerets oriented ventrally, tan with narrow bowed black longitudinal stripes running about 3/2 the length and a thicker black lateral band running all around, dorsal area with several long sparsely placed setae (Fig. 1a, b, c, d); male with single orange scutum laterally and posteriorly (Fig. 1d), female without sclerite around spinnerets.

##### Male palp

Median apophysis (MA) with multiple lobes. Embolus (E) long, flexible, runs distally from median apophysis, then turns to run in proximal direction (Fig. 4). Cymbium (CB) covers most of retrolateral face of bulb, with dark tooth-like processes (CT) near proximal dorsal part.

##### Vulva

**Figure 2.**
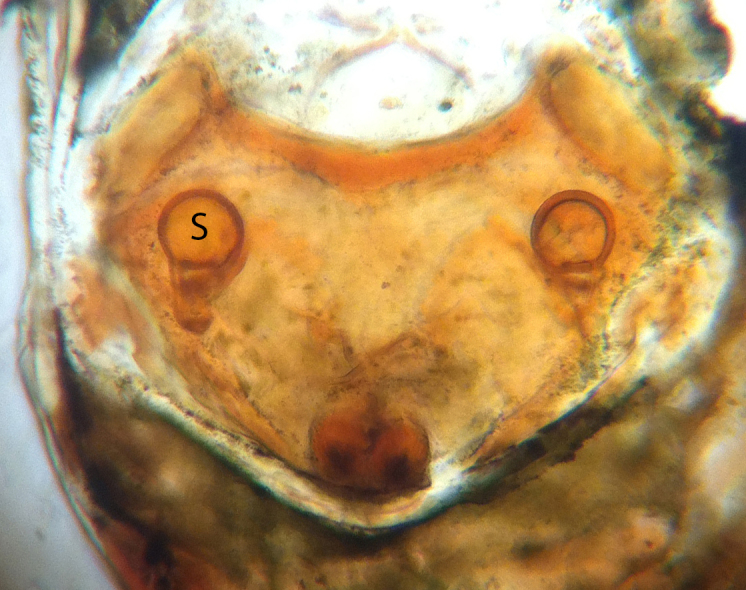
*Crassignatha danaugirangensis* sp. n., vulva, dorsal view, cleared in palm oil. S, spermatheca.

Scape small and rounded. Round spermathecae separated by more than three times their diameter (Fig. 2).

##### Measurements

Male: Total length 0.7; carapace length 0.3, width 0.3, height 0.2. Female: Total length 0.9; carapace length 0.3, width 0.3, height 0.2.

#### Diagnosis

Distinctive abdominal coloration separates this from all other *Crassignatha* species (Fig. 1a, b, c, d); only this species and *Crassignatha haeneli* Wunderlich, 1995 have longitudinal bands on abdomen (a broad light band on an otherwise dark gray abdomen in *Crassignatha haeneli*, Wunderlich 1995). Distinguished from *Crassignatha haeneli* by the unicolor legs (banded in *Crassignatha haeneli*, Wunderlich 1995); male further distinguished from *Crassignatha haeneli* by the presence of an abdominal scutum in the male (Fig. 1d; absent in *Crassignatha haeneli*, Wunderlich 1995). Male distinguished from all except *Crassignatha haeneli* by the tibia II clasping spur consisting of only a single ventral macroseta (Fig. 1f; 2–4 in other species). Female distinguished from all other *Crassignatha* species by the widely spaced spermathecae (separated by more than three times their diameter in this species, not more than 1.5 times their diameter in other species).

#### Etymology

Named for the Danau Girang Field Centre, the type locality for this species.

The taxonomic authority for this species is attributed to all authors of this publication. In accordance with ICZN Recommendation 51C (International Commission on Zoological Nomenclature 1999), this species may be referred to as *Crassignatha danaugirangensis* Miller et al., 2014, provided the full citation of this publication appears in the bibliography or elsewhere in the referring work. At a time when scientific research in general is becoming more collaborative and multidisciplinary, it should not be surprising to find increasing numbers of authors responsible for nomenclatural acts. Arguably, the convention in zoology of referencing the authors of a taxonomic name (ICZN Article 51) rather than the source publication is anachronistic in contemporary multidisciplinary, collaborative science (Costello 2009).

#### Distribution

Known only from the forest of the Danau Girang Field Centre.

#### Ecology

**Figure 3.**
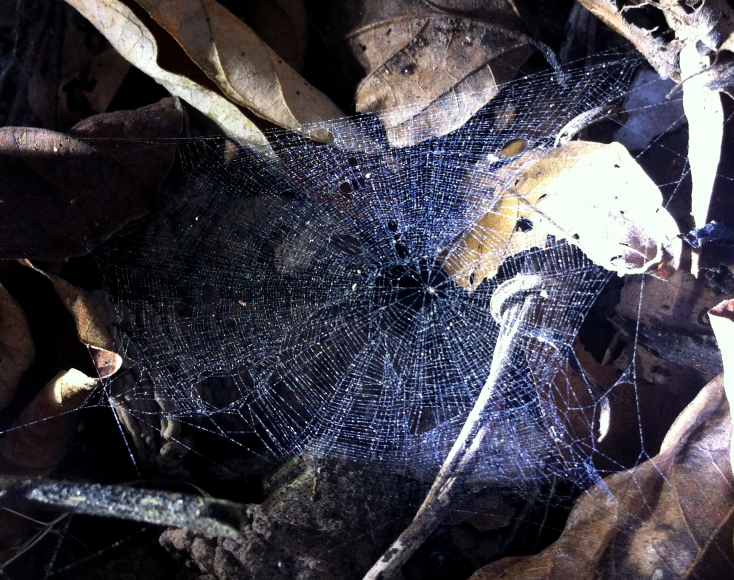
*Crassignatha danaugirangensis* sp. n. in web after being dusted with corn starch.

**Figure 4.**
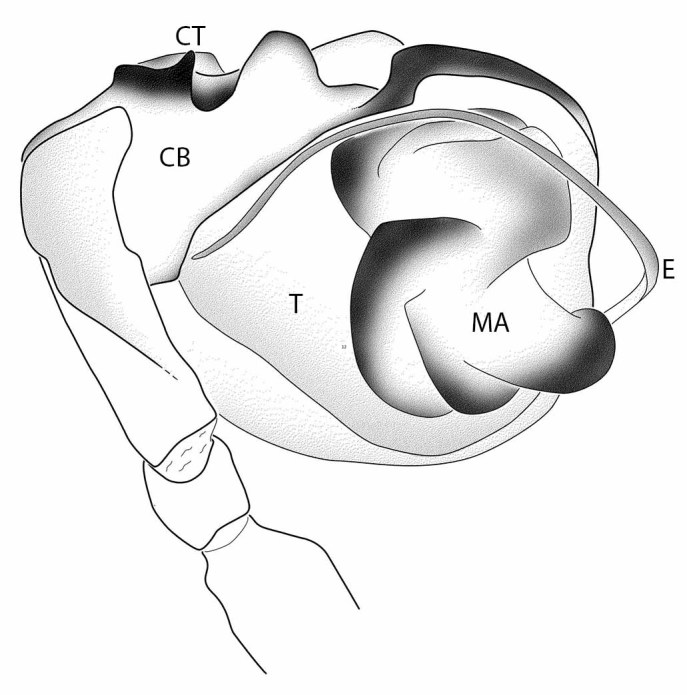
*Crassignatha danaugirangensis* sp. n., left male palp, prolateral view. CB, cymbium; CT, cymbial tooth; E, embolus; MA, median apophysis; T, tegulum.

This species builds a horizontal orb web approximately 4 cm in diameter, close to the ground (Fig. 3).

## Analysis

A total of 79 adult ground-web-building spiders were collected during the plot survey. Overall ground web spider density was significantly higher in the riparian forest (5.2 per m^2^) compared to the other habitats investigated (1.3 and 1.75 per m^2^ in riverine forest and oil palm plantation, respectively; ANOVA with Tukey's pairwise comparisons, *p* < 0.05). *Crassignatha danaugirangensis* sp. n. was the most abundant species found with 38 adults (48.1%, 3.8 per m^2^). The next most abundant species overall was a member of the Hahniidae, with 7 individuals (8.9%). *Crassignatha danaugirangensis* was found only in the riparian forest plots. Plotless sampling in the oil palm plantation failed to find any *Crassignatha*. See also Suppl. material 1 for raw morphospecies abundance data by plot.

## Discussion

**Figure 5.**
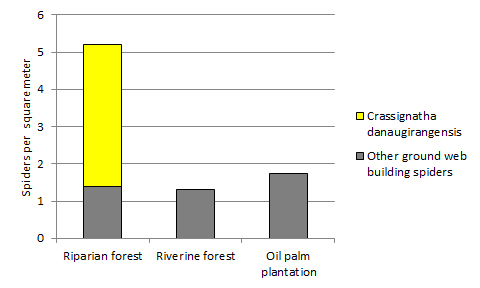
Density of *Crassignatha danaugirangensis* sp. n. as a proportion of all adult ground-web-building spiders sampled from 1 m^2^ plots in riparian forest, riverine forest, and oil palm plantation. See Suppl. material 1 for raw morphospecies abundance data.

Periodic inundation is a regular feature of the forest at Danau Gurang. Some of the low lying forests, including botanical plots 2 and 3, were flooded one to two weeks prior to this study. In addition to *Crassignatha danaugirangensis*, the community of ground-web builders at Danau Girang includes linyphiids, mysmenids, theridiosomatids, and hahniids. The difference in ground web spider abundance found in the recently flooded and unflooded botanical plots can be attributed to the exclusive presence of *Crassignatha danaugirangensis* in the unflooded plots (Fig. 5, Table 1); reanalysis of the data without *Crassignatha* erases the difference in ground-web spider density between the habitat types (ANOVA, *p* = 0.88). This suggests that *Crassignatha* is particularly sensitive to forest disturbance, whether this is due to natural causes like flooding or profound anthropogenic causes such as palm oil agriculture.

As a coda to the field course, we organized a *Crassignatha* blitz. Students, instructors, and DGFC staff were organized into teams of two and sent to various points around the trail network. Each team used a sock to contain a quantity of corn starch, which when lightly tapped over the leaf litter, suffices as a puffer. Teams were trained to identify *Crassignatha* webs, and were asked to search for webs in their assigned area for 10 minutes. Teams were asked to photodocument a sample of the webs they observed, especially any observations about which they were not entirely certain. The results show that *Crassignatha danaugirangensis* is widespread along the DGFC trail network, but is rare if not absent in the most recently flooded forest patches. This raises the question: does *Crassignatha danaugirangensis* actually prefer riparian forest over riverine forest, or is this the case only shortly after an inundation event? To answer this question, further study of this species will be required. But in the absence of a durable and accessible taxonomy, it becomes virtually impossible to accumulate knowledge about a species between studies conducted by independent researchers. Until now, *Crassignatha danaugirangensis* was one of countless undescribed species. The ecological sensitivity of this species suggested by our brief study calls for further observation and monitoring. It also raises new questions about the inundation forest ecology of the invertebrates at a research station normally focused on some of the world's most charismatic vertebrates. The taxonomic description presented here and made accessible to all via an open access cybertaxonomic journal will facilitate this activity.

### Epilogue

According to the investigation of Fontaine et al. (2012), the average time from collection to description of a new species is 21 years, something that we, with an unusually high level of cooperation throughout the research cycle, accomplished within one month. Cybertaxonomic enhancements, such as the simultaneous appearance of the species description on the Encyclopedia of Life and the occurrence data on the Global Biodiversity Information Facility (GIBF), increase the routes available to access some of the key data presented here. While we don't generally endorse applying such a frenetic pace to the meticulous and detailed scholarship of taxonomy, we do support a multifaceted approach creatively employing traditional and cybertaxonomic tools to reduce the number of undescribed species and increase the availability of fundamental taxonomic information.

## Supplementary Material

Supplementary material 1 Spider morphospecies sampled from riparian forest, riverine forest, and oil palm plantation Data type: Structured sampling data
Brief description: Adult ground-web-building spider morphospecies sampled from 1 m^2^ plots in the Danau Girang botanical plots and nearby Hilco Estate oil palm plantation. The number of 1 m^2^ plots in each site is given as *n*. Botanical plots 1 and 4 are riparian forest habitat, plots 2 and 3 are riverine forest habitat subject to frequent inundation.
File: oo_6233.xls Jeremy A. Miller, Jennie Lilliendahl Burmester, Lot van der Graaf

XML Treatment for Crassignatha

XML Treatment for Crassignatha danaugirangensis

## Figures and Tables

**Figure 1a.**
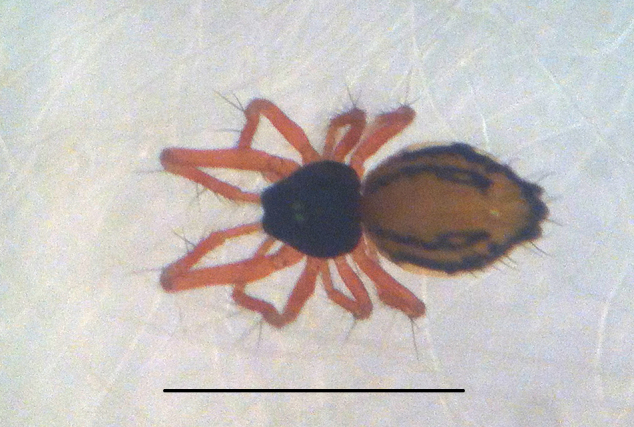
*Crassignatha danaugirangensis* sp. n., female, dorsal view. Scale bar 1 mm.

**Figure 1b.**
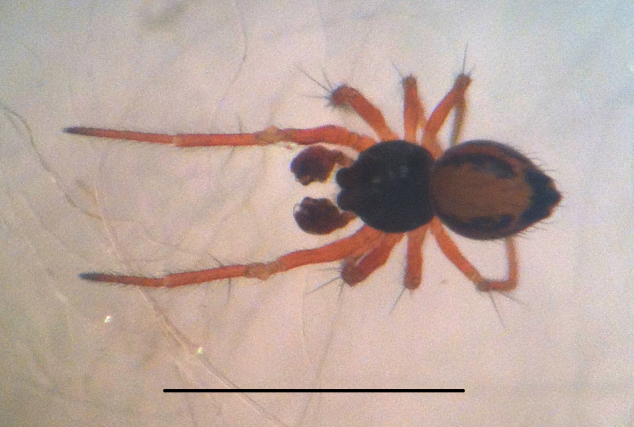
*Crassignatha danaugirangensis* sp. n., male, dorsal view. Scale bar 1 mm.

**Figure 1c.**
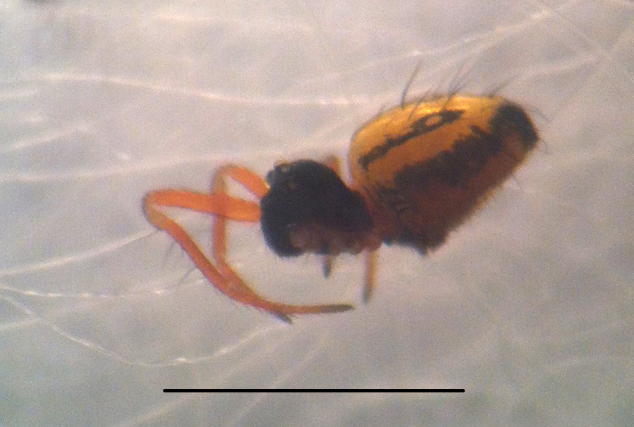
*Crassignatha danaugirangensis* sp. n., female, lateral view, left side legs removed. Scale bar 1 mm.

**Figure 1d.**
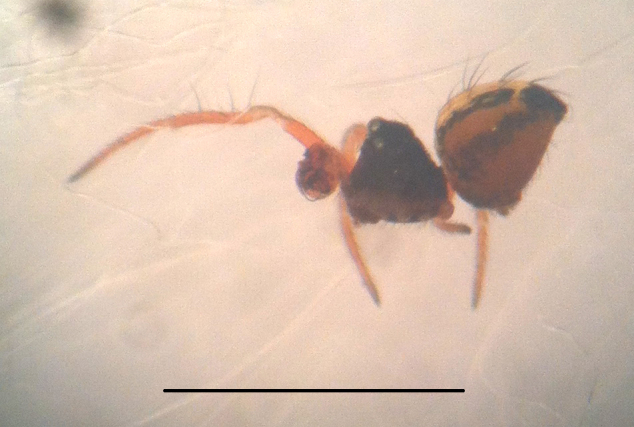
*Crassignatha danaugirangensis* sp. n., male, lateral view, left side legs removed. Scale bar 1 mm.

**Figure 1e.**
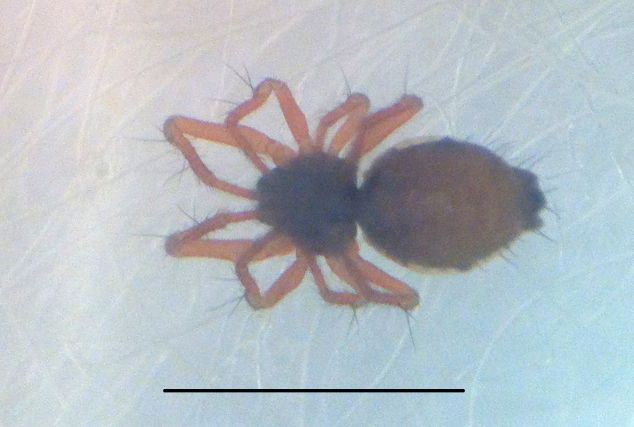
*Crassignatha danaugirangensis* sp. n., female, ventral view. Scale bar 1 mm.

**Figure 1f.**
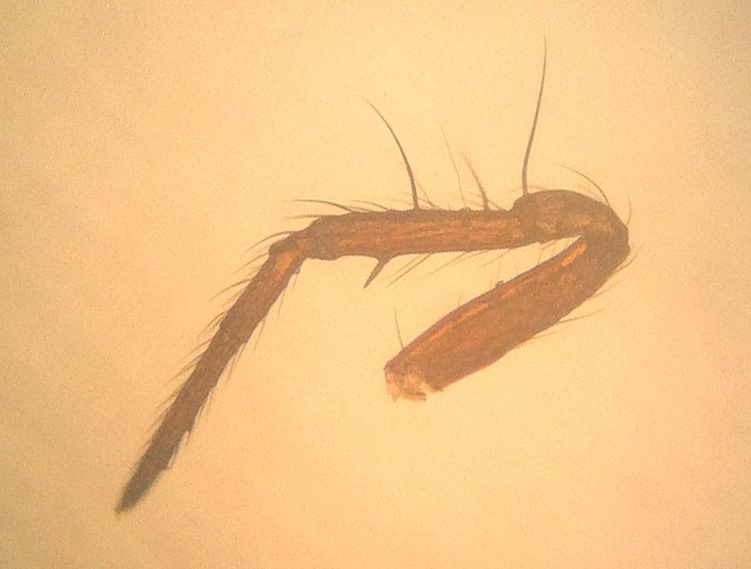
*Crassignatha danaugirangensis* sp. n., male, left leg II, retrolateral view.

**Table 1.** Environmental data and results from the plot survey. Tree species richness and total number of trees (tree count) in four 0.25 ha plots are reported. Number of oil palms per 0.25 ha was estimated using Google Earth (images dated 2009), and tree species richness was assumed to be approximately 1. Sample sizes in parentheses refer to the number of 1 m^2^ samples within each botanical plot. Spider data are adults per square meter ± standard error. See also Suppl. material 1 for raw morphospecies abundance data.

	Riparian forest		Riverine forest		Oil palm plantation
	Botanical plot 1 (*n* = 6)	Botanical plot 4 (*n* = 4)	Botanical plot 2 (*n* = 6)	Botanical plot 3 (*n* = 4)	(*n* = 8)
Tree species	51	45	31	32	1
Tree count	179	164	219	178	25
Spiders	6.5 ± 1.7	3.25 ± 1.7	1.5 ± 0.5	1.0 ± 0.4	2.3 ± 1.0
*Crassignatha danaugirangensis* sp. n.	5.2 ± 1.1	1.8 ± 1.8	0	0	0
